# 

**Published:** 1887-03

**Authors:** 


					﻿Kaftmict Uv. »W. O$iet, at	Tex-j*. <w ,sifcwn«t-C’»w» 5FU!
Vol. II,	MARCH, x88;.

- ■‘^s? f *
_ a ««?JA'IsW -SBf!
MIM sfi &.
Villi,’
g iHStfttl
4 ~ ;loOt
Subscription, S2.00 a Year, in Advance. — Single Copies, Twenty-fiveCen s.
F E. DANIEL.. M. D., Editor and Publisher, AUSTIN^ TEXAS. \
Northern Ofece. t«&7 Filbert street. Phiiadrl^hia.
___	’	t "’’L J
EuGE.\Ej/o\ Bokckmann, S:kam Book and Joh Pr!ni£R, Acs n-;f Texas?'
				

